# miR-199a Targeting PNRC1 to Promote Keratinocyte Proliferation and Invasion in Cholesteatoma

**DOI:** 10.1155/2021/1442093

**Published:** 2021-11-16

**Authors:** Lihui Yao, Wenjing Zhang, Jian Zheng, Xing Lu, Fan Zhang

**Affiliations:** Department of Otolaryngology, The First Affiliated Hospital of Zhengzhou University, Zhengzhou 450052, China

## Abstract

**Introduction:**

miR-199a has been reported as an oncogene of various cancers. However, the biological function and regulatory mechanism of miR-199a in keratinocytes of cholesteatoma are still unclear.

**Methods:**

Detection by qRT-PCR was conducted on miR-199a's expression in both thirty pairs of cholesteatoma tissues and normal skins. For characterizing the function of miR-199a, this research adopted transwell assay, wound healing assay, and CCK8 assays. Under the support of qRT-PCR, efforts were made to investigate the relative expression of candidate target genes. Moreover, the evaluation of the targeting relationship between miR-199a and the candidate target gene was conducted with the dual-luciferase reporter assay.

**Results:**

The upregulation of miR-199a was found in cholesteatoma tissues, which facilitated the proliferation, migration, and invasion of HaCaT cells, while its downregulation caused opposite results.

**Conclusions:**

The findings of the present research offer more insights into the molecular mechanism of cholesteatoma progression.

## 1. Introduction

Middle ear cholesteatoma, instead of a tumor, refers to a mass of squamous epithelium in the middle ear that produces keratin. According to the theoretical mechanism of pathogenesis, middle ear cholesteatoma can be divided into congenital cholesteatoma and acquired cholesteatoma [1]. The former only accounts for 2-4% of all cases and mainly occurs in children aged from 4 to 6 [2]. In contrast, the latter can also happen to children and adults [3, 4].

Specifically, in addition to the clinical symptoms including ear pain, ear discharge, and hearing loss, cholesteatoma has serious complications such as meningitis, encephalitis, epidural abscess, or sigmoid sinus thrombosis [5]. In terms of the pathological features of the cholesteatoma epithelium, there are also out-of-control proliferation, migration, aberrant differentiation, and aggressiveness [6]. The pathogenesis of cholesteatoma otitis media, commonly seen in otorhinolaryngology, has always been a research hotspot. Substantial research has found that acquired cholesteatoma displayed a close correlation with continuous infection and inflammation in the middle ear. Also, several investigators reported that tumor-related genes and markers of proliferation such as CDH18, CDH19, and ID4 were upregulated in cholesteatoma specimens [7–10]. Despite a large amount of research efforts, the underlying cellular and molecular mechanisms of acquired cholesteatoma remain unclear. It should be noted that keratinocytes are the main cell type of cholesteatoma.

MicroRNAs (miRNAs) refer to small and noncoding RNAs able to inhibit the translation of mRNA and/or intervene in the degradation of mRNA to regulate the posttranscriptional expression [11]. Furthermore, they function significantly to regulate proliferation, differentiation, migration, and apoptosis of cells [12]. In previous studies, numerous miRNAs such as miR-let-7a, miR-203a, and miR-21 were determined to be correlated with the pathogenesis of cholesteatoma [11, 13, 14]. However, the role of microRNA-199a (miR-199), an extensively studied cancer-promoting microRNA, in cholesteatoma has not attracted researchers' attention. miR-199, a highly conserved miRNA family, is associated with various tumors. Between two types of miR-199, miR-199a and miR-199b, more research focus has been put on the former [15], finding its important role in enhancing the chemosensitivity of hepatocellular carcinoma (HCC) [16]. The decreased expression of miR-199a was identified as a feature of advanced renal cell cancer [17], and miR-199a was found to facilitate the progress of colorectal cancer as a tumor-promoting gene in colorectal cancers [18]. Yet, studies of miR-199a as a multifunctional microRNA with regard to the ear are relatively scarce. Among them, one just reported its essential effect on the function and development of the inner ear for vertebrates [19]. However, its specific role in the pathogenesis of cholesteatoma is still unclear although its expression profile is similar to that of a metastatic tumor and chronically inflamed tissue [20].

In the present study, we found that the expression levels of miR-199a were correlated with cholesteatoma and determined as upregulated. Correspondingly, the overexpression of miR-199a in cell experiments promoted abnormal proliferation and migration of HaCaT cells. Our results suggest that the high expression of miR-199a is probably an influence factor resulting in the development of cholesteatoma. Therefore, these studies can provide a new perspective for the functions of miR-199a in HaCaT cells and its regulatory mechanism in the progression of cholesteatoma. Further research on miR-199a can possibly provide a potential diagnostic and therapeutic target for the treatment of cholesteatoma.

## 2. Materials and Methods

### 2.1. Patients and Specimens

The approval of the Ethics Committee of the First Affiliated Hospital of Zhengzhou University (Ethical Review No. 2020-KY-225) for the whole research, as well as all involved patients' (or their parents') written informed consent, was obtained before the research. Cholesteatoma tissues and matched normal postauricular skin tissues [11, 21, 22] were obtained from cholesteatoma patients conformed by pathologists in the First Affiliated Hospital of Zhengzhou University from March 2020 to June 2020. The collected specimens were sent to the laboratory for immediate surgery. Then, liquid nitrogen was used to freeze them, which were stored at –80°C for RNA extraction.

### 2.2. RNA Preparation and Quantitative Real-Time PCR

Under the instructions of the manufacturer, TRIzol Reagent (Invitrogen, Carlsbad, CA, USA) was adopted to purify total RNA, respectively, from cholesteatoma and normal postauricular skin tissues. The measurement of optical density at 260 nm was conducted to quantify the extracted RNA. Then, guided by the manufacturer's protocol, HiScript® III RT SuperMix for qPCR (+gDNA wiper) (Vazyme, Nanjing, China) or miRNA first-strand cDNA synthesis kit (by stem-loop) (Vazyme, Nanjing, China) was used for the reverse transcription of 1 *μ*g RNA to cDNA in a 20 *μ*l reaction system. Moreover, under the manufacturer's instructions, miRNA Universal SYBR qPCR Master Mix (Vazyme, Nanjing, China) or ChamQ Universal SYBR qPCR Master Mix (Vazyme, Nanjing, China) was applied to a quantitative real-time polymerase chain reaction (qPCR), where an 18 *μ*l reaction system was added with 2 *μ*l cDNA sample. The PCR reaction of miR-199a and U6 was performed by an initial denaturation at 95°C for 5 min, followed by 40 cycles at 95°C for 10 sec, 60°C for 35 sec, and elongation at 95°C for 15 sec, 60°C for 60 sec, and 95°C for 15 sec. The PCR cycles of mRNA were run for an initial denaturation at 95°C for 30 sec, followed by 40 cycles at 95°C for 10 sec, 60°C for 30 sec, and elongation at 95°C for 15 sec, 60°C for 60 sec, and 95°C for 15 sec. The calculation was performed on the expression of miRNAs to obtain threshold cycle (CT) values, where miRNAs or mRNA expression levels were normalized by the 2^−ΔΔCT^ method, and U6 or GAPDH was taken as the endogenous control. Each reaction was repeated three times to calculate the mean value with SD. The primer sequences used are listed in Table 1.

### 2.3. Cell Culture and miRNA Transfection

HaCaT cells were purchased from ATCC (EK-Bioscience, Shanghai, China), and DMEM cell culture medium (GIBCO, Germany) with 10% fetal bovine serum (FBS) (Coolaber, Beijing, China) was applied. HaCaT cells were stored in an incubator of 5% CO_2_ at 37°C. Mycoplasma testing has been done. The transfection involved the specimens with the miR-199a inhibitor, miR-199a mimics, or a negative control miRNA (GenePharma, Shanghai, China) and supported by RFect^PM^ small nucleic acid transfection reagent for primary cells (Baidai, Changzhou, China). RFect^PM^ small nucleic acid transfection reagent for primary cells (Baidai, Changzhou, China) is a proprietary formulation for transfecting small nucleic acids (miRNAs, siRNA, and antisense RNA) into a wide range of primary adherent cells. RFect^PM^ can be added directly to cells in culture medium, in the presence or absence of serum/antibiotic. HaCaT cells (2.0 × 10^6^ cells/well) were seeded into 6-well plates (2 ml/well) and cultured at 37°C (5% CO_2_) until 70–80% confluence. DMEM cell culture medium (GIBCO, Germany) was used to dilute RFect^PM^ and miRNAs. miRNAs and RFect^PM^ were mixed at room temperature, and finally, the miRNA-RFect^PM^ mixture was directly added to the corresponding plate of the above 6-well plates. Cells were collected at 24, 36, and 48 h after the transfection for qRT-PCR to calculate the efficiency of transfection. Then, the best transfection conditions were determined for subsequent experiments.

### 2.4. Cell Proliferation Assay

The proliferation of HaCaT cells was measured with the CCK8 assays for the evaluation of miR-199a's effects. Cells after transfection were seeded in 96-well culture plates at 2 × 10^3^ cells per well in a 5% CO_2_ incubator at 37°C for 5 consecutive days. Subsequently, at 0 h, 24 h, 48 h, 72 h, and 96 h, each well was added with 10 *μ*l CCK8 (Dojindo, Japan) for 3 h at 37°C. At 450 nm, a microplate reader was then used to get absorbance values. Notably, all experiments were repeated no less than three times.

### 2.5. Wound Healing Assay

The 6-well plates were used to culture HaCaT cells in the logarithmic growth phase until 80%-90% confluence. Then, a 200 *μ*l sterile pipette tip was used to scratch these cells, and phosphate-buffered saline (PBS) was used to wash them two times for removing nonadherent cells. After scraping, the distance of migration was accessed at 0 h and 48 h. The images were captured under the microscope with a digital camera.

### 2.6. Transwell Assay

The invasive ability of HaCaT cells was evaluated by using a transwell chamber (24-well insert, 8 mm pore size, Corning, New York, USA) in a transwell assay. The transwell upper chamber covered with a Matrigel containing 200 *μ*l of serum-free DMEM cell culture medium (GIBCO, Germany) was adopted to resuspend 3 × 10^5^ cells. DMEM cell culture medium of 650 *μ*l, together with 20% FBS, served as the lower chamber. Then, 48 h later, we fixed migrated cells in 4% paraformaldehyde for 30 min, followed by 30 min of staining with crystal violet (Solarbio, Beijing, China). At last, five fields were selected randomly, and a light microscope (magnification, ×40) and Image analysis software (ImageJ, MD, USA) were used to count the number of migrated HaCaT cells.

### 2.7. Target Prediction

The online biological databases TargetScan (https://www.targetscan.org/), TarBase (http://microrna.gr/tarbase/), and miRDB (http://mirdb.org/) were applied to the prediction of miR-199a targets. The intersection of three gene sets was identified using a Venn diagram.

### 2.8. Luciferase Assay

How miR-199a interacted with the 3′-UTRs of PNRC1 was predicted by the wild-type (WT) or mutant (MUT) 3′-UTRs of PNRC1. The two forms of 3′-UTR of PNRC1 and miR-199a mimic were transfected together into 293T cells purchased from ATCC (EK-Bioscience, Shanghai, China) under the supports of X-tremeGENE HP DNA Transfection Reagent (ROCHE, Basel, Switzerland) and then inserted into the GV272 vector (GENE, Shanghai, China) in 24-well plates. Additional luciferase analysis was performed on the cells collected after forty-eight hours of cotransfection which were harvested with the aid of a Dual-Luciferase® Reporter Assay System (Promega, Madison, WI, USA).

### 2.9. Statistical Analysis

GraphPad Prism 8.0 software (La Jolla, CA, USA) was adopted to determine the results of statistical analyses. All experiments were performed no less than three times in the same procedure. The mean ± standard deviation (SD) was used to express data, and Student's unpaired *t*-test was applied to the analysis of the significance of differences between two groups, where statistical significance was defined as *P* < 0.05.

## 3. Results

### 3.1. Expression of miR-199a in Cholesteatoma and HaCaT Cells

**Figure 1:**
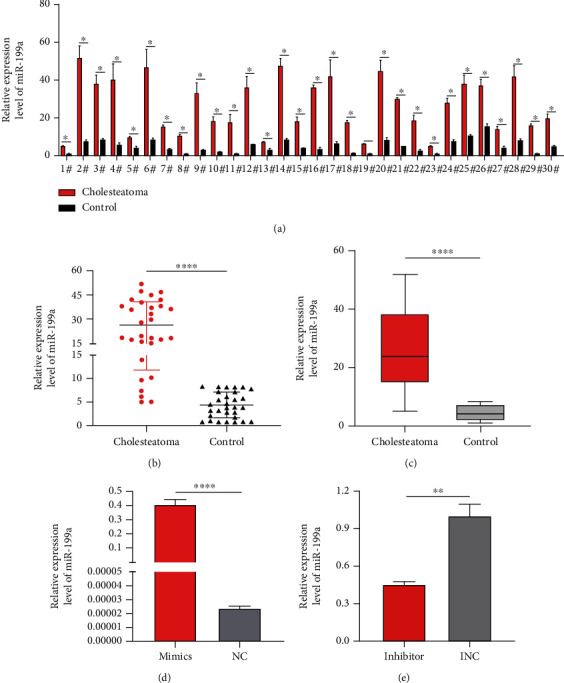
miR-199a's expression in cholesteatoma and HaCaT cells. (a) Gene expression measured by qPCR in 30 pairs of cholesteatoma tissues and normal retroauricular skin (^∗^*P* < 0.05). (b) miR-199a's expression in 30 pairs of cholesteatoma tissues and normal retroauricular skin detected by qPCR (^∗∗∗∗^*P* < 0.0001). (c) The distribution of 30 pairs of tissues detected by qPCR (^∗∗∗∗^*P* < 0.0001). (d) Efficiency of overexpressed miR-199a measured by qPCR in transfected HaCaT cells. ^∗∗∗∗^*P* < 0.0001 vs. control. (e) Efficiency of miR-199a knockdown tested by qPCR in transfected HaCaT cells. ^∗∗^*P* < 0.01 vs. control.

For the comparison of miR-199a between cholesteatoma and normal retroauricular skin, we collected 30 pairs of cholesteatoma and normal retroauricular skin tissues for later qRT-PCR, finding that miR-199a was significantly upregulated in cholesteatoma tissues compared to the normal retroauricular skin tissues (Figures 1(a)–1(c)). The results of qPCR of the HaCaT cells transfected with miR-199a mimics, miR-199a inhibitor, and the negative control are shown in Figures 1(d) and 1(e). All subsequent cell experiments were performed under this transfection condition.

### 3.2. Role of miR-199a in HaCaT Cell Proliferation, Migration, and Invasion

**Figure 2:**
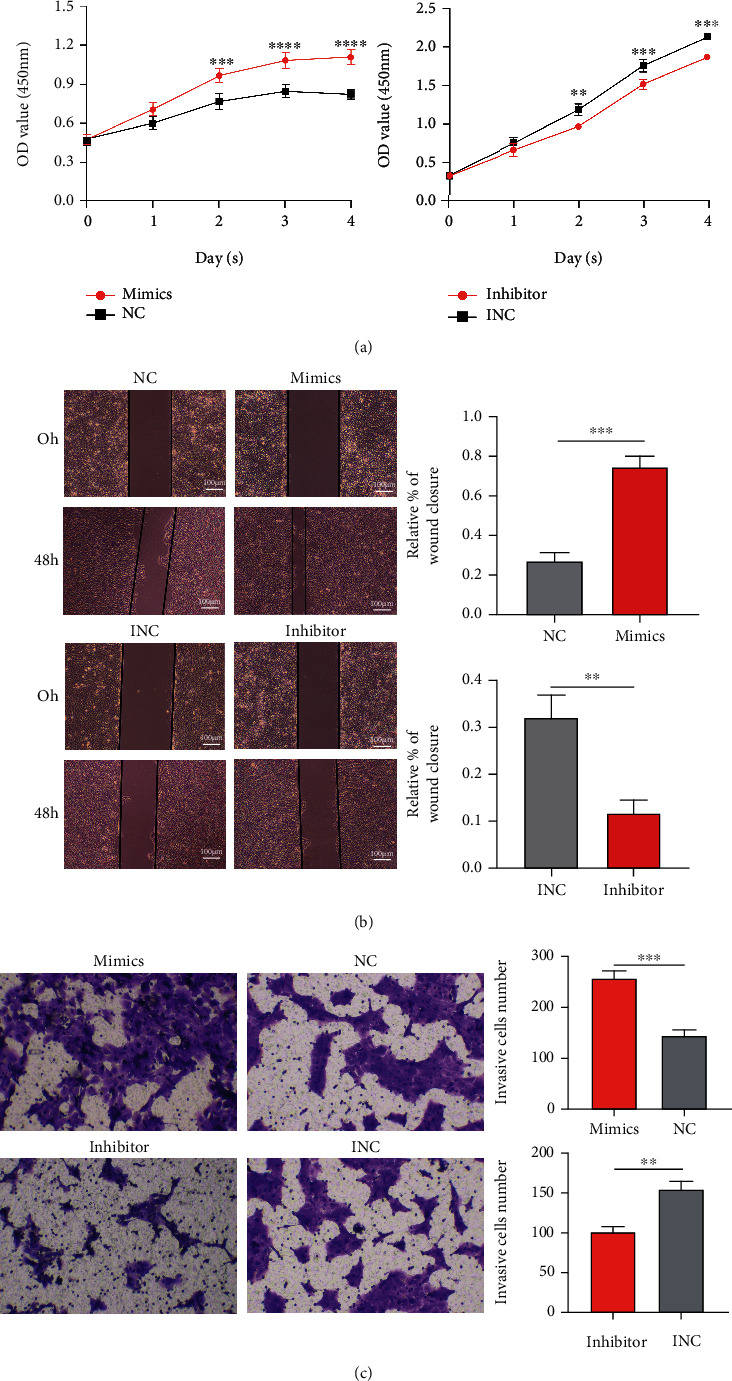
Effects of miR-199a on proliferation, migration, and invasion of HaCaT cells. ^∗∗^*P* < 0.01, ^∗∗∗^*P* < 0.001, and ^∗∗∗∗^*P* < 0.0001. (a) The role in promoting the proliferation of HaCaT cells. (b) The role in promoting the migration of HaCaT cells. Migration index (%) = [(the initialized width of the scratch) − (the final width of the scratch)]/(the initialized width of the scratch). Scale bar represents 100 *μ*m. (c) The role of miR-199a in promoting the invasion of HaCaT cells detected by a Matrigel invasion assay.

As mentioned above, miR-199a mimics and inhibitor were found to be effectively and successfully transfected into HaCaT cells (Figures 1(d) and 1(e)). The CCK8 assay indicated that HaCaT cell proliferation was highly promoted by the upregulation of miR-199a but inhibited by its downregulation (Figure 2(a)). The results of the wound healing assay and transwell assay, as shown in Figures 2(b) and 2(c), implied the positive role of miR-199a in the migration and invasion of cholesteatoma keratinocytes in vitro.

### 3.3. Target Genes Associated with miR-199a

**Figure 3:**
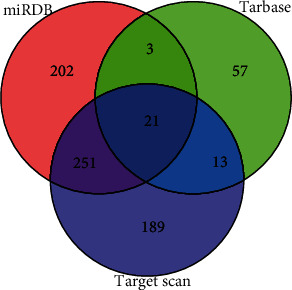
The overlap RNA among the three datasets in the Venn diagram.

**Figure 4:**
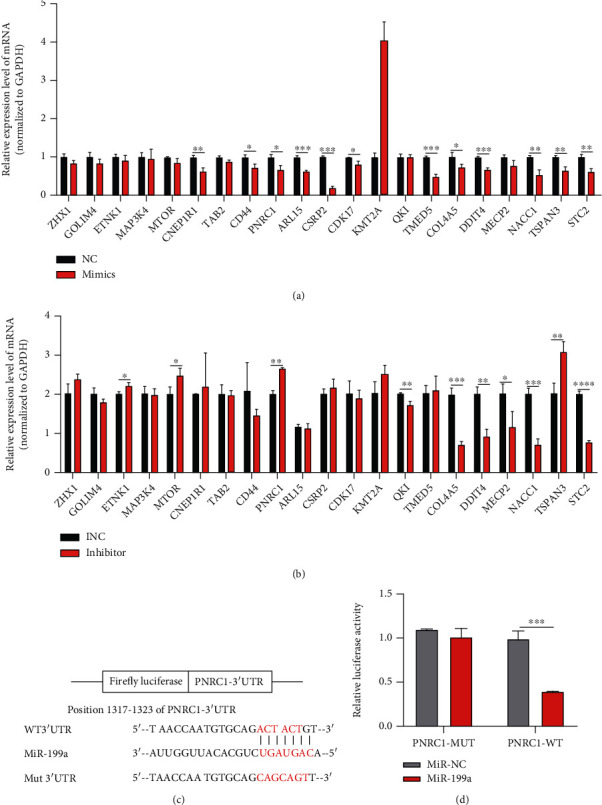
Negative regulation of PNRC1 by miR-199a. ^∗^*P* < 0.05, ^∗∗^*P* < 0.01, ^∗∗∗^*P* < 0.001, and ^∗∗∗∗^*P* < 0.0001. (a) Expression of candidate target mRNAs detected in cells transfected with miR-199a mimics. (b) Expression of candidate target mRNAs tested in cells transfected with the miR-199a inhibitor. (c) The 3′-UTR of PNRC1 containing a putative binding site for miR-199a and a mutation of the 3′-UTR of PNRC1. (d) The luciferase assay showing the role of miR-199a in inhibiting PNRC1–3′-UTR luciferase activity of HaCaT cells.

The TargetScan (https://www.targetscan.org/), miRDB (http://mirdb.org/), and TarBase (http://microrna.gr/tarbase/) databases were applied for the prediction of miR-199a's target genes. From a Venn diagram that displays the overlap of the three databases, 21 common target genes were found (Figure 3). Then, we detected the expression of these candidate 21 target mRNAs by qPCR in cells transfected with miR-199a mimics or inhibitor and then selected and labeled the target mRNAs with statistical significance. The crosscomparison revealed that PNRC1 was strongly negatively correlated with miR-199a (Figures 4(a) and 4(b)).

### 3.4. miR-199a Negatively Regulated PNRC1 by Directly Binding to Its 3′-UTR

We used a luciferase assay to validate how miR-199a correlated with PNRC1 through the design of a mutation of the 3′-UTR of PNRC1 (PNRC1-MUT, Figure 4(c)).  Figure 4(d) presents that miR-199a upregulated by the mimic caused a significant reduction in PNRC1-WT's luciferase activity but not in that of PNRC1-MUT, implying the binding of miR-199a with the 3′-UTR of PNRC1.

### 3.5. PNRC1 Was Low Expressed in Cholesteatoma

**Figure 5:**
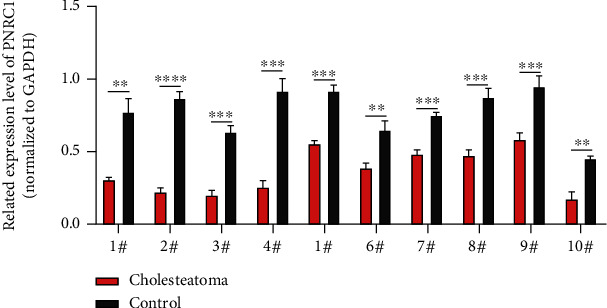
Expression of PNRC1 measured by qPCR in 10 pairs of cholesteatoma and normal retroauricular skin. ^∗∗^*P* < 0.01, ^∗∗∗^*P* < 0.001, and ^∗∗∗∗^*P* < 0.0001.

Compared to the paired retroauricular skin sample, ten cholesteatoma tissues displayed significant downregulation of PNRC1 (Figure 5). This result was just contrary to the high expression of miR-199a in cholesteatoma, indicating a negative regulatory relationship between miR-199a and PNRC1.

## 4. Discussion

Cholesteatoma is known as a severe chronic middle ear disease that occurs in the middle ear, eventually causing adverse complications with rapid growth and bone resorption [23]. It accounts for 0.5–1.8% of all brain tumors [24]. Elucidating the underlying molecular mechanism for the progression of cholesteatoma will help provide more treatment options for this disease. Further study can be explored in the keratinocyte cell culture model [25].

miRNAs have vital functions in regulating cellular activities. Over the past years, substantial studies have confirmed the involvement of miRNA in the pathogenesis of acquired cholesteatoma [11, 14, 26]. Notably, miR-199a has attracted wide attention. Functional research has identified mature miR-199a's crucial role in maintaining normal homeostasis and regulating disease pathogenesis [15].

In the current study, qRT-PCR was performed to examine the expression levels of miR-199a in cholesteatoma and adjacent normal tissues, revealing the upregulation of miR-199a in cholesteatoma tissues. These results demonstrate the oncogenic role of miR-199a in the development of cholesteatoma. Furthermore, the gain and loss function experiments of miR-199a were carried out to observe how the physiological process of keratinocytes was affected by miR-199a. Our study discovered that the growth rate of HaCaT cells reduced when knocking out miR-199a via the miR-199a inhibitor. The metastasis potential of HaCaT cells decreased, which was confirmed by the transwell assay and wound healing assay. The overexpression of miR-199a in HaCaT cells produced the opposite results. Here, we identified the expression pattern and function of miR-199a in cholesteatoma.

Furthermore, we explored the downstream of miR-199a. Based on the predicting results from three databases, we conducted correlation analysis between 21 target genes and miR-199a in HaCaT cells transfected with miR-199a mimics or inhibitor. We could find that both TSPAN3 and PNRC1 showed a strong correlation with miR-199a. We only chose PNRC1 for the following study, because miR-199a has a cancer-promoting effect in our study; then, we need to find a target gene that is negatively correlated with miR-199a. In general, we would like to find a tumor suppressor gene as a target gene, and PNRC1 is just the right mRNA among the 21 candidate mRNAs [27, 28]. However, it would be of an area of interest for further research on TSPAN3. Interestingly, we found that PNRC1's expression was downregulated in cholesteatoma tissues and PNRC1's expression increased in HaCaT cells transfected with the miR-199a inhibitor and PNRC1's expression decreased in HaCaT cells transfected with miR-199a mimics at the mRNA level. The dual-luciferase assay indicated that miR-199a had the great potential to bind with PNRC1. As a result, we concluded that miR-199a exerts its function by targeting PNRC1. However, more assays concerning the function of PNRC1 in cholesteatoma will be needed to verify this regulation. In addition, we noticed that the PNRC1's expression had not changed in HaCaT cells transfected with the miR-199a inhibitor or mimics at the protein level. We speculated that PNRC1 was regulated by miR-199a via protein translation, posttranslational modification, or degradation mechanisms according the previous research, and Chen et al. pointed out that the protein expression level may not necessarily be related to mRNA expression level at sometimes [29], which supported our results. Anyway, more assays will be needed to explore this.

In summary, the present research first confirmed miR-199a as a tumor promoter in cholesteatoma. Its biological function in HaCaT cells was verified by knockdown and overexpression experiments, together with the preliminary discussion of its mechanism. We found that miR-199a promoted keratinocyte proliferation, migration, and invasion in cholesteatoma by directly targeting PNRC1. Our results may help improve the understanding of the pathogenesis of cholesteatoma and present some guiding significance for the development of treatment strategies for cholesteatoma. Nevertheless, more research effort is still needed to determine the regulatory mechanisms of miR-199a for understanding its roles in pathogenesis of cholesteatoma. Furthermore, the research on the relationship between cholesteatoma and microRNAs is still in the exploratory stage. In the future, with the constant advancement in the experimental techniques and development of the theoretical basis, more groundbreaking pioneering research results related to miRNA and cholesteatoma will appear to bring good news to patients with cholesteatoma in the world.

## Figures and Tables

**Table 1:** PCR primer information.

Gene	Gene ID	Description	5′-primer-3′
miR-199a	406976	Stem-loop	GTCGTATCCAGTGCAGGGTCCGAGGTATTCGCACTGGATACGACTAACCA
miR-199a	406976	F	GCGCGACAGTAGTCTGCACAT
		R	AGTGCAGGGTCCGAGGTATT
U6	/	F	CTCGCTTCGGCAGCACA
		R	AACGCTTCACGAATTTGCGT
TSPAN3	10099	F	TCACAGAAGTTGTTGTAGTGGT
		R	CCCAGTCTGAGTAGTTGTGAAT
STC2	8614	F	TCATCAAAGACGCCTTGAAATG
		R	CAGCAAGTCCTTGAAATGGATC
KMT2A	4297	F	GCCAAGAAAAGAAGTTCCCAAA
		R	CTGCATTCTCCTGCTTATTGAC
TMED5	50999	F	AAAAGTGGGCACATACAAACTC
		R	TCTTCAAACAGACTCTTCAGCA
COL4A5	1287	F	CCTCACATTCCTCCTAGTGATG
		R	CTTTGTCACCTTTCACTCCTTG
DDIT4	54541	F	GATGCCTAGCCAGTTGGTAAG
		R	CTAAACAGCCCCTGGATCTTG
MTOR	2475	F	GAGATACGCTGTCATCCCTTTA
		R	CTGTATTATTGACGGCATGCTC
TAB2	23118	F	TTAGGCAGCAAAGGAACATCTA
		R	TACGACCAGTCTGGATATTTGG
PNRC1	10957	F	GCAGGATTCTGTTTCATCTGAC
		R	ATTTTCAACAGTGCTTCCCATC
CSRP2	1466	F	TCACGATGAAGAGATCTACTGC
		R	AGTGTTTGGATTTGTTGTAGGC
CDK17	5128	F	CAGGTGTTTGTCTCAGAAATCG
		R	GATAAGGAAGCTCTACGAGACC
QKI	9444	F	GATCAGACAAATACAGACCGCT
		R	GTAGGGGTACTCATAGGGTGTA
ZHX1	11244	F	CCTATGTTTGTGTCGAATGCAA
		R	TGCTCTGCATTCTCCTCTTTAA
ETNK1	55500	F	CATGGCCAATTACATCCACG
		R	GATGCTCCTCCTGATCCTGAA
MAP3K4	4216	F	GAATGCTGCTGAAATCTACAGG
		R	TAACAGACCTCCTGATTTCGTC
CNEP1R1	255919	F	GCAGGCGGAAGATCTCAAGGC
		R	GAAGCATTCTCCAGCGTCCAGTAG
CD44	960	F	GGGAGTCAAGAAGGTGGAGCAAAC
		R	GCCAAGAGGGATGCCAAGATGATC
ARL15	54622	F	TGGAGCCGCTACTACCAAGGATC
		R	CTGTGGATGCTGAAGAGCTGAGTG
MECP2	4204	F	ACCACCATCACCACCACTCAGAG
		R	GACGCTGCTGCTCAAGTCCTG
NACC1	112939	F	ACCAGCCCAGGCACCTCAAG
		R	CTCGCCACCATCCTCCTCCTC
GOLIM4	27333	F	CTGCTGCTGACCGTCGTGTTC
		R	TCCTGGTGCTGCTGGTACTTGAG

## Data Availability

The raw data of this study are available from the corresponding author on reasonable request.
